# A study on the mechanisms of teachers’ academic emotions and motivational beliefs on learning engagement in the context of online training

**DOI:** 10.3389/fpsyg.2023.1255660

**Published:** 2023-09-18

**Authors:** Dongdong Zhang, Siyuan Gao, Liling Ren

**Affiliations:** ^1^Faculty of International Studies, Henan Normal University, Xinxiang, China; ^2^Department of Education, Henan Normal University, Xinxiang, China

**Keywords:** online learning engagement, academic emotions, precision training, educational meta universe, control-value theory, expectancy value theory

## Abstract

**Introduction:**

In the context of digital transformation of education, online training is one of the important ways for teachers to improve their professionalism and promote the quality of education. However, studies have shown that teachers’ online training suffers from insufficient learning engagement and other problems, so it is crucial to explore the factors influencing teachers’ learning engagement and their mechanisms of action in the context of online training.

**Methods:**

Taking 589 teachers who participated in online training as the research subjects, the study used the methods of survey research and statistical analysis to explore the influence mechanism of teachers’ academic emotions and motivational beliefs on online learning engagement based on the dual perspectives of control value theory and expectancy-value theory.

**Results:**

The study found that: (1) positive-high arousal academic emotions, training self-efficacy, and training task value significantly and positively predicted online learning engagement, respectively; (2) negative-high arousal and negative-low arousal academic emotions significantly and negatively predicted online learning engagement; (3) training self-efficacy and training task value mediated the relationship between positive-high arousal academic emotions, negative-high arousal academic emotions, negative-low arousal academic emotions and online learning engagement, respectively.

**Discussion:**

The study concluded that by creating an immersive learning environment based on the educational meta universe, personalized and precise training based on big data and adaptive technologies, and establishing a multi-dimensional and three-dimensional online learning support service system, which can effectively improve teachers’ online learning engagement and enhance their online training quality and effectiveness.

## 1. Introduction

In April 2022, the Ministry of Education and other eight departments issued the document “Plan for Strengthening Teachers in Basic Education in the New Era, “which pointed out that “it is necessary to deepen the reform of precision training, innovate online and offline hybrid training models, and comprehensively improve the quality of teacher training.” The EU’s Action Plan for Digital Education (2021–2027) released in September 2020 emphasizes “the need to improve digital skills and competencies for digital transformation,” which shows that digital transformation in the education sector is imperative, and online training will play an important role in the long term as an important cornerstone of education quality improvement (European Commission, 2022). Since the implementation of two rounds of the National Primary and Secondary School Teachers’ Information Technology Application Capacity Enhancement Project, more than 13 million teachers nationwide have been trained online, with more than 1.3 billion cumulative learning visits, and the important role of online training has become increasingly evident, especially since the outbreak of the COVID-19 (The Chinese Ministry of Education, 2022). Online learning engagement is a key indicator of the effectiveness of online learning (Ronimus et al., 2014), and studies have explored the mechanisms of motivational beliefs, motivational regulation (Zhang et al., 2020), etc. on online learning engagement or online learning academic achievement (Liang et al., 2023) from the perspectives of self-determination theory, social cognitive theory, and self-regulated learning. Meanwhile, emotion as an important non-cognitive psychological factor is an important predictor variable of online learning engagement, in which positive academic emotion promotes learners’ learning engagement and negative academic emotion hinders learning engagement (Reeve and Shin, 2020), however, some studies also suggest that negative academic emotion increases learners’ learning engagement (Turner and Schallert, 2001). At present, there are few researches on teachers’ academic emotion in China, and there are not enough empirical studies on teachers’ online learning engagement. The relationship between teachers’ academic emotion and online learning engagement in online training lacks empirical support.The dual role of teachers as students and adult online learners when participating in online training, how academic emotions contribute to online learning engagement, and whether task value, as an important construct of motivational beliefs, mediates the relationship between academic emotions and online learning engagement need to be further investigated.

Control-value theory is an integrative theory for predicting individual behavioral engagement, providing a comprehensive and systematic framework for analyzing academic emotions in learning, classroom, and examination contexts, while expectancy-value theory is an important motivational theory for predicting individual behavioral choices. Therefore, from the dual perspectives of control value theory and expected value theory, this study combined with relevant research status in the field of education, and sorted out the influencing factors of academic emotion and online learning engagement, examines the relationship and mechanism between teachers’ academic emotions, motivational beliefs and online learning engagement, in order to provide theoretical support and empirical evidence for improving the quality of online training. Specifically, it can provide empirical evidence for teacher educators to make relevant decisions, provide references for implemutors and designers of teacher training programs and teacher training resources, and ultimately improve the effectiveness of teacher training and the quality of teachers.

## 2. Research basis

### 2.1. Control-value theory of academic emotions

Pekrun et al. (2002) first defined the concept of academic emotion in 2002: the various emotional experiences experienced by learners in the three contexts of learning, classroom, and examination, directly pointing to academic activity or academic results themselves The control-value theory of academic emotions suggests that individuals’ academic emotions in learning, classroom, and test-related processes arise from their own control and value assessments of their learning. According to the control-value theory, the individuals’ judgment of their own abilities and value assessment are important factors influencing the individual’s academic emotions and determining whether the learner’s academic emotions are aroused or not (Pekrun et al., 2007). The control-value theory is universally applicable and bi-directionality of the relationship. Universally applicable means that between academic emotions and its influencing factors is applicable to all groups of learners of different socio-cultural backgrounds, ages and genders. Bi-directionality of the relationship in that not only do control and value assessments determine the arousal of academic emotions, but academic emotions will in turn influence the subsequent control and value assessments of learners (Pekrun, 2006).

Academic emotions include enjoyment, hope, pride, anger, anxiety, shame, relaxation, disappointment, and boredom. Based on control-value theory, the nine academic emotions are classified into four categories according to pleasure and arousal: positive-high arousal (enjoyment, hope, pride), negative-high arousal (anger, anxiety, shame), positive-low arousal (relaxation), and negative-low arousal (disappointment, boredom). Due to the specificity of online learning environment and teachers as adult learners, teachers mainly experience three types of academic emotions: positive-high arousal academic emotions (enjoyment, pride), negative-high arousal academic emotions (anxiety, shame), and negative-low arousal academic emotions (disappointment, boredom) in online learning contexts (Zhan, 2016). Based on the online teacher training context, this study explores the mechanism of academic emotion’s influence on learning engagement from the above three dimensions.

### 2.2. Research related to academic emotions and online learning engagement

Learning engagement is an important indicator of learners’ learning behavior and academic achievement (Ronimus et al., 2014). Christenson et al. (2012) argue from a psychological perspective that individual online learning engagement includes three dimensions: cognitive, behavioral, and affective, and that there are links between the three dimensions Sinha from the sociological perspective analyzes the current state of online learners’ learning engagement and points out that online learning engagement includes cognitive, behavioral, social, and conceptual-effect inputs, of which social input refers to learners’ interaction with others in the learning process, and conceptual-effect input refers to learners’ use of knowledge and technology tools to solve problems (Sinha et al., 2015). The key feature of online learning is to encourage active collaboration among learners and between teachers and students (Swartzwelder et al., 2019). Therefore, social interaction input is an important dimension of online learning engagement, and this study concludes that teachers’ online learning engagement includes four dimensions: cognitive, behavioral, affective, and social interaction.

Researches related to academic emotions in the context of online learning has shown that academic emotions are strongly related to online learning engagement. For example, one study found that positive emotions can help EFL students stay more focused on learning tasks and promote their engagement (Hiver et al., 2021). And in another study, positive emotions promoted students’ interest and thinking and helped them focus on the learning task by increasing their awareness of and engagement with the task (Dewaele et al., 2016). However, the effect of academic emotions on engagement in online learning is more complex, as Turner and Schallert (2001) found in their study that negative-high arousal academic emotions increased learners’ engagement in learning by increasing learners’ extrinsic motivation to avoid failure. The above suggests that academic emotions are the important influence on online learning engagement, and the role of different types of academic emotions on online learning engagement varies. Therefore, this study proposes the following hypothesis:

*H1*: In the context of online training, positive-high arousal academic emotions had a significant positive effect on online learning engagement.

*H2*: In the context of online training, negative-high arousal academic emotions had a significant positive effect on online learning engagement.

*H3*: In the context of online training, negative-low arousal academic emotions had a significant negative effect on online learning engagement.

### 2.3. Mediating role of motivational beliefs between academic emotions and online learning engagement

Learners’ motivational beliefs are critical to their maintaining high levels of engagement in learning (Bandura, 1982). One of the important theories explaining individual motivation for achievement is the expectancy-value theory, which suggests that individual’s expectation of success and task value are important predictors of their learning behavior and achievement (Bandura, 1986; Liem et al., 2008). The modern EVT was proposed by Eccles et al. (1983) and has had a huge impact on the field of achievement motivation. Modern expected value theory holds that self-efficacy and task value are important factors to predict individual achievement behavior, choice and persistence. Learning engagement is the direct expression of individual achievement behavior, choice and persistence, so the expected value theory has a strong guiding role for this study. Here we mainly use Wang and Liou (2018) current expected value theory to divide the motivational belief dimension. Specifically, motivational beliefs include self-concept, intrinsic value and utility value. Self-concept refers to the individual’s perception of the difficulty of a learning task and self-assessment of their own abilities; intrinsic value refers to an individual’s inner interest in learning activities; and utility value refers to the usefulness of the task for the individual’s future development (Wigfield and Eccles, 2000, 2019). The connotation of self-concept is similar to that of self-efficacy, which refers to an individual’s judgment of whether he or she has the ability to complete a task, and both emphasize an individual’s perception of their own ability and prediction of future motivation, emotion, and achievement (Bong and Skaalvik, 2003), Previous studies related to online learning engagement have not made a distinction between the two and have mostly adopted the concept of self-efficacy (Zhang and Liu, 2019). Expected value theory holds that self-efficacy and task value are important factors to predict individual achievement behavior and they are also two important concepts of motivational belief (Phan, 2009). Therefore, the motivational beliefs in this study used training self-efficacy, training task value as the research dimensions. Training self-efficacy refers to teachers’ perceptions of their ability to complete the training tasks when participating in online training, and training task value refers to teachers’ judgments of whether online training is beneficial to their future development.

The predictive role of task value and self-efficacy on engagement in online learning has been verified. For example, Gorozidis and Papaioannou (2014) found a positive predictive effect of teachers’ intrinsic motivation on their willingness to participate in training. Durksen et al. (2017) found a significant positive relationship between teachers’ self-efficacy and learning engagement in their study. Zhang et al. (2020) investigated the mechanism of the relationship between teachers’ motivational beliefs and learning engagement in online training by combining the perspectives of expectancy-value theory and self-regulated learning, and found that teachers’ perceived task value positively predicted learning engagement. Therefore, the following hypotheses were proposed in this study:

*H4*: Teacher training self-efficacy has a significant positive effect on online learning engagement in online training contexts.

*H5*: Teacher training task value has a significant positive effect on online learning engagement in online training contexts.

Self-efficacy and task value, two of the more important concepts in motivational beliefs, mediate the relationship between academic emotions and online learning engagement. In their study, Lin et al. (2020) found that positive academic emotions, negative outcome directed activity academic emotions and learning engagement were significantly and positively correlated, and academic self-efficacy partially mediated the relationship between positive academic emotions, negative activity directed academic emotions and learning engagement. As task value is another important dimension of motivational belief, whether there is a mediating role between academic emotion and online learning engagement needs to be further explored, so this study proposes the following hypotheses:

*H6/H7/H8*: Teacher training self-efficacy in online training contexts is mediated between academic emotions (positive-high arousal, negative-high arousal, negative-low arousal) and online learning engagement.

*H9/H10/H11*: In the context of online training, teacher training task value mediates between academic emotions (positive-high arousal, negative-high arousal, negative-low arousal) and online learning engagement.

**Figure 1:**
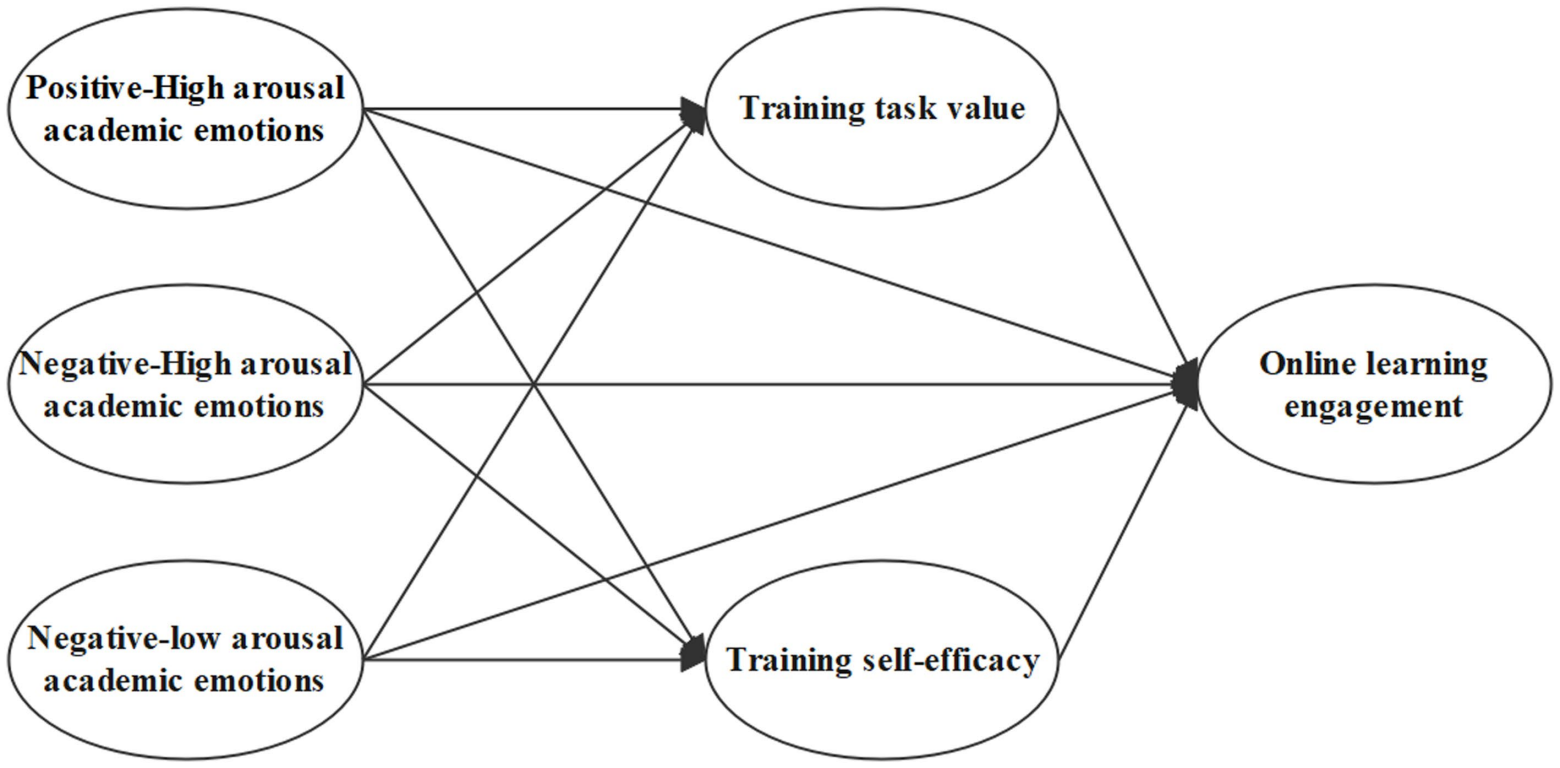
Theoretical model construction.

Based on the above analysis, this study constructs the model shown in Figure 1 to investigate the role and paths of teachers’ academic emotions and motivational beliefs on online learning engagement in the context of online training.

## 3. Methodology

### 3.1. Research subjects

This study was a randomized sampling cross-sectional survey. The research area is China, as well as the study subjects were teachers who participated in online training, and this study collected research data by sending research questionnaires to them through the Questionnaire Star platform. A total of 589 questionnaires were collected, 92 invalid questionnaires with less than 120 s response time and all the same options were deleted, and 497 valid questionnaires were obtained, with a recovery rate of 84.4%. Among them, 147 were males and 350 were females, accounting for 29.6 and 70.4% respectively, and those aged 20 ~ 30, 30 ~ 40, 40 ~ 50, and over accounted for 16.1, 31.6, 43.9, and 8.2%, respectively.

### 3.2. Instruments

#### 3.2.1. Online learning engagement scale

The online learning engagement scale used in this study was modified from the online learning engagement questionnaire developed by Sun and Dixson, and was divided into four dimensions: cognition (5 items), behavior (5 items), emotion (4 items), and social interaction (4 items),with a total of 18 items (Dixson, 2010; Sun and Rueda, 2012). The study used the Likert 7-point scale, from 1 to 7 representing “completely disagree” to “completely agree,” and the higher the total score of each dimension, the higher the corresponding level of teacher engagement.In this study, the original questions were modified by replacing the contextual condition of “online learning” with “online training,” and the modified items were: “I am able to follow the rules and regulations of the online training,” “I am able to concentrate at all times while attending the online training,” etc. The Cronbach coefficients for each dimension of the modifiedscale are 0.89, 0.79, 0.85, and 0.89, respectively.

#### 3.2.2. Academic emotions scale

The academic emotions measurement instrument used in this study was the Pekrun Academic Emotion Questionnaire, which measured six types of academic emotions in the context of teachers’ online training learning activities, divided into three dimensions: positive-high arousal (13 items), negative-high arousal (13 items), and negative-low arousal (10 items), with a total of 36 items. The study used a 7-point Likert scale, ranging from 1 to 7, representing “completely disagree” to “completely agree,” with higher total scores indicating higher levels of teachers’ corresponding academic emotions. In this study, the original items were modified to add the context of “online training,” and the modified items were: “I am looking forward to learning more in the next online training,” “The content of the online training is interesting, so I am willing to spend more time I am willing to spend more time learning because of this.” The Cronbach coefficients for each dimension of the modifiedscale are 0.80, 0.71, and 0.91, respectively.

#### 3.2.3. Motivational belief scale

The motivational beliefs in this study were divided into two dimensions, training self-efficacy and training task value, selected from the study of Artino and Mccoach (2008), in which 4 items were trained in self-efficacy and 5 items were valued by training tasks, for a total of 9 items. The study used a 7-point Likert scale, ranging from 1 to 7, representing “completely disagree” to “completely agree,” with higher scores indicating higher levels of training self-efficacy or training task value. In this study, the original questions were modified by replacing the term “online courses” with “online training.” Revised questions such as “The online training provided me with a lot of practical information” and “Even though the content was very difficult, I felt confident that I could understand the learning in the online training.” The Cronbach’ alpha for each dimension of the modified scale were 0.70 and 0.87, respectively.

**Table 1:** Correlation coefficient matrix.

	M	SD	PH	NH	NL	TSE	TTV	OAE
PH	5.95	0.86	1					
NH	3.15	1.31	−0.380**	1				
NL	2.64	1.19	−0.461**	0. 598**	1			
TSE	5.57	0.93	0.625**	−0.267**	−0.347**	1		
TTV	5.76	0.94	0.641**	−0.348**	−0.461**	0.563**	1	
OAE	5.66	0.81	0.761**	−0.405**	−0.485**	0.717**	0.804**	1

PH, NH, NL, TSE, TTV, and OAE represent positive-high arousal academic emotions, negative-high arousal academic emotions, negative-low arousal academic emotion, training self-efficacy, training task value, and online learning engagement, respectively.

**p < 0.01.

### 3.3. Data processing

In this study, questionnaires were distributed through the Questionnaire Star online platform, the data screening and collation was carried out by Excel2010, the model correction and reliability and validity analysis were performed by Amos24.0, the correlation analysis of each dimension was performed using SPSS25.0.Multiple mediation models can take on two forms: parallel or chain mediation (Hayes, 2009), and we used the SPSS plug-in program Process23.0 to test the parallel mediation effect, as it can handle more complex models with multiple mediators, multiple moderators, and moderated mediators (Liang et al., 2023).

## 4. Research results

### 4.1. Descriptive statistics and correlation analysis

The results of Pearson coefficients for teachers’ academic emotions, motivational beliefs, and online learning engagement in online training are shown in Table 1. There were significant positive correlations between positive-high arousal academic emotions, training self-efficacy, training task value, and online learning engagement, while negative-high arousal and negative-low arousal academic emotions were all correlated with training self-efficacy, training task value, and online learning engagement. On this basis, the influence mechanism of academic emotions and motivational beliefs on online learning engagement can be further explored.

**Table 2:** Reliability and validity test.

Variables	Factor load range	Convergent validity		Distinct validity					
		CR	AVE	PH	NH	NL	TSE	TTV	OAE
PH	0.68 ~ 0.80	0.725	0.679	0.824					
NH	0.60 ~ 0.83	0.914	0.681	−0.380**	0.852				
NL	0.76 ~ 0.92	0.722	0.573	−0.461**	0. 598**	0.757			
TSE	0.62 ~ 0.74	0.871	0.692	0.625**	−0.267**	−0.347**	0.832		
TTV	0.81 ~ 0.86	0.808	0.585	0.641**	−0.348**	−0.461**	0.563**	0.765	
OAE	0.63 ~ 0.92	0.888	0.668	0.761**	−0.405**	−0.485**	0.717**	0.804**	0.817

PH, NH, NL, TSE, TTV, and OAE represent positive-high arousal academic emotions, negative-high arousal academic emotions, negative-low arousal academic emotion, training self-efficacy, training task value, and online learning engagement, respectively.

**p < 0.01.

### 4.2. Measurement model reliability test

In order to verify whether the hypothesis is valid, this study tested the reliability of the measurement model for each variable, and the results are shown in Table 2. The results show that the range of factor loadings for each variable is higher than 0.6, indicating good convergent validity of the questionnaire factors; the composite reliability (CR) values are higher than 0.7, indicating good construct reliability of the measurement model; the average variance extracted (AVE) is higher than 0.5 (Hair et al., 2021), indicating good convergent validity of the measurement model. The square root of any two variables AVE is greater than their correlation coefficients, indicating good discriminant validity between the measurement models.

**Table 3:** Results of path hypothesis test.

**Paths**	**Coeff**	**SE**	***p*-value**	**Assumptions**
Positive-high arousal of academic emotions → Online learning engagement	0.277	0.028	<0.001	Hypothesis 1 holds
Negative-high arousal academic emotions → Online learning engagement	−0.069	0.015	<0.001	Hypothesis 2 is not valid
Negative-low arousal academic emotions → Online learning engagement	−0.071	0.017	<0.001	Hypothesis 3 holds
Training self-efficacy → Online learning engagement	0.239	0.024	<0.001	Hypothesis 4 holds
Training task value → Online learning engagement	0.398	0.024	<0.001	Hypothesis 5 holds
Positive-high arousal of academic emotions → Training self-efficacy	0.673	0.038	<0.001	
Negative-high arousal academic emotions → Training self-efficacy	−0.190	0.031	<0.001	
Negative-low arousal academic emotions → Training self-efficacy	−0.271	0.033	<0.001	
Positive-high arousal of academic emotions → Training task value	0.699	0.038	<0.001	
Negative-high arousal of academic emotions → Training task value	−0.250	0.030	<0.001	
Negative-low arousal academic emotion → Training task value	−0.364	0.032	<0.001	

### 4.3. A test of the mediating effect of training self-efficacy and training task value between academic emotions and online learning engagement

**Figure 2:**
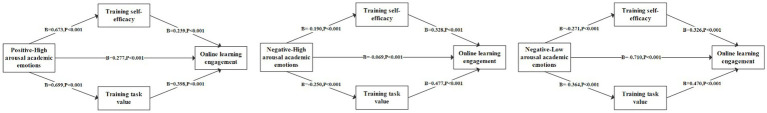
Path model of the influence of academic emotion on online learning engagement.

In this study, three parallel mediation models were developed with three types of academic emotions as independent variables, online learning engagement as dependent variables, and self-efficacy and task value as mediating variables, respectively and the specific path diagram is shown in Figure 2. As shown in Table 3, positive-high arousal academic emotions (*β* = 0.277, *p* < 0.001), training self-efficacy (*β* = 0.239, *p* < 0.001), and training task value (*β* = 0.398, *p* < 0.001) significantly and positively predicted online learning engagement, and negative-high arousal (*β* = −0.069, *p* < 0.001), negative-low arousal academic emotions (*β* = −0.71, *p* < 0.001) significantly and negatively predicted online learning engagement, hypotheses 1, 3, 4, and 5 were valid, and hypothesis 2 was not valid. Table 4 shows that training self-efficacy has a significant partially mediated effect between positive-high arousal, negative-high arousal, and negative-low arousal academic emotions and online learning engagement, respectively, with 95% confidence intervals of [0.117, 0.220], [−0.093, -0.038], and [−0.128, −0.058], respectively, none of which contain 0. The mediated effect values are 0.161, −0.062, −0.088, accounting for 22.5, 24.7, and 26.6% of the total effect, respectively, and hypotheses 6, 7, and 8 hold; The 95% confidence intervals for the training task value between positive-high arousal, negative-high arousal, and negative-low arousal academic emotions and online learning engagement are [0.215, 0.346], [−0.162,−0.085], [−0.227, −0.124], none of which contained 0. The mediating effect values were 0.278, −0.120, and − 0.171, which accounted for 38.9, 47.8, and 51.7% of the total effect, respectively, indicating that the training task value partially mediated the effect between the three types of academic emotions and learning engagement, and hypotheses 9, 10, and 11 were valid.

**Table 4:** A test of the mediating effect of motivational beliefs between academic emotions and online learning engagement.

**Effect**	**Paths**	**Effect value**	**Standard error**	**Percentage of total effect**	***P*-value**	**95% CI**		**Assumptions**
						**LLCL**	**ULCL**	
Direct effect	Positive-high arousal of academic emotions → Online learning engagement	0.277	0.028	38.7%	<0.001	0.222	0.331	
	Negative-high arousal academic emotions → Online learning engagement	−0.069	0.015	27.5%	<0.001	−0.098	−0.040	
	Negative-low arousal academic emotions → Online learning engagement	−0.071	0.017	21.5%	<0.001	−0.105	−0.037	
Indirect effects	Positive-high arousal of academic emotions → Training self-efficacy → Online learning engagement	0.161	0.023	22.5%	<0.001	0.117	0.220	Hypothesis 6 holds
	Positive-high arousal of academic emotions → Training task value → Online learning engagement	0.278	0.033	38.9%	<0.001	0.215	0.346	Hypothesis 9 holds
	Negative-high arousal academic emotions → Training self-efficacy → Online learning engagement	−0.062	0.014	24.7%	<0.001	−0.093	−0.038	Hypothesis 7 holds
	Negative-high arousal academic emotion → Training task value → Online learning engagement	−0.120	0.019	47.8%	<0.001	−0.162	−0.085	Hypothesis 10 holds
	Negative-low arousal academic emotions → Training self-efficacy → Online learning engagement	−0.088	0.018	26.6%	<0.001	−0.128	−0.058	Hypothesis 8 holds
	Negative-low arousal academic emotions → Training task value → Online learning engagement	−0.171	0.026	51.7%	<0.001	−0.227	−0.124	Hypothesis 11 holds
Total effect	Positive-high arousal of academic emotions → Online learning engagement	0.715	0.027		<0.001	0.662	0.769	
	Negative-high arousal academic emotions → Online learning engagement	−0.251	0.025		<0.001	−0.301	−0.200	
	Negative-low arousal academic emotions → Online learning engagement	−0.331	0.027		<0.001	−0.383	−0.278	

## 5. Conclusion and discussion

Based on the control-value theory of academic emotions and the expectancy-value theory of achievement motivation, this study explored the relationship between teachers’ academic emotions and online learning engagement in the context of online training and tested the mediating role of motivational beliefs. The findings indicated that training self-efficacy and training task value mediated the relationship between all three types of academic emotions (positive-high arousal, negative-high arousal, and negative-low arousal) and online learning engagement, respectively, which further validated the important roles of academic emotions and motivational beliefs in learners’ learning. While control-value theory suggests that control assessment and value assessment are proximal factors in arousal of academic emotions, this study verifies the path of academic emotions on learners’ “value assessment” and online learning engagement from the reverse direction, which enriches control-value theory and provides evidence to support the quality improvement of online training.

### 5.1. Academic emotions are significant predictor of online learning engagement

Consistent with previous research (Mulualem et al., 2022; Nauta and Lehman, 2022), the results of the study indicated that positive-high arousal academic emotions had a significant positive predictive effect on online learning engagement, and negative-high arousal and negative-low arousal academic emotions had a significant negative predictive effect on online learning engagement. The results of this study support the control-value theory path hypothesis that positive academic emotions predict higher academic achievement, indicating that learners increase their learning engagement, while negative-high arousal and negative-low arousal academic emotions decrease online learning engagement by weakening learners’ motivation levels and taking up cognitive resources. In the positive-high arousal academic emotions state, learners are able to maintain their attention and use more advanced learning strategies during learning process, while the negative-high arousal academic emotions state tends to make learners use simple and mechanical learning strategies. And the negative-low arousal academic emotions state ties up learners’ cognitive resources, making them unable to concentrate and avoid learning tasks, which leads to the use of superficial learning strategies and is not conducive to deeper information processing and processing. Therefore, it is important to focus on the factors associated with academic emotions in teacher training to enhance teachers’ online learning engagement.

### 5.2. Training self-efficacy and training task value are significant predictors of online learning engagement

The results of the study indicated that teachers’ training self-efficacy and the value of training tasks in online training significantly and positively predicted online learning engagement, respectively. Expectancy-Value theory suggests that motivational beliefs are powerful predictors of individual behavioral choices and academic achievement, and that learners are more likely to exhibit positive learning behaviors if they have higher levels of self-efficacy and more meaningful perceived value for the current learning task. Similarly, self-regulation theory states that learners with higher levels of self-efficacy and task value are more effective at motivational regulation, which in turn motivates learners to give more cognitive input into the learning process (Cleary and Zimmerman, 2004). The role of training self-efficacy and training task value in influencing online learning engagement has also been validated in previous studies (Zhao and Cao, 2023). For example, Watt et al. (2014) found that teachers’ self-efficacy significantly predicted their professional engagement in a study of pre-service teachers’ professional engagement and professional development vision.

### 5.3. Training self-efficacy and training task value mediate the relationship between academic emotions and online learning engagement

The results of the study showed that training self-efficacy and training task value in online training partially mediated the relationship between each of the three types of academic emotions (positive-high arousal, negative-high arousal, and negative-low arousal) and online learning engagement, respectively, suggesting that academic emotions can indirectly act on online learning engagement through motivational beliefs. Control assessment, value assessment, and academic emotions are bidirectional in that the learner’s assessment of control and value is the main determinant of whether academic emotions are aroused, but academic emotions also affect control assessment and value assessment in turn, i.e., it has an impact on self-efficacy and task value. Emotions and motivational beliefs interact in cognitive processes and jointly influence learning outcomes. Cognitive processes include the use of cognitive strategies and self-regulated learning (Kim et al., 2014), positive academic emotions motivate learners to enhance internal motivation and use flexible high-level learning strategies (e.g., refinement, metacognition, etc.) (Artino and Jones, 2012), and negative-high arousal, negative-low arousal academic emotions take cognitive resources away from the learning process, making it difficult for learners to use high-level learning strategies, again reducing motivation and hindering learning engagement.

## 6. Strategies and suggestions

This study verified the mediating role of motivational beliefs between academic emotions and online learning engagement from the reverse paths of academic emotions on control assessment and value assessment, and revealed the paths of academic emotions and motivational beliefs on online learning engagement with different degrees of pleasure and arousal through mediating effect analysis, which theoretically confirmed and further extended control-value theory and expectancy-value theory. Meanwhile, the findings of this study have important implications for enhancing online learning engagement in teachers’ online training.

### 6.1. To create an immersive online training environment based on the educational meta universe

Social cognitive theory points out that the dynamic interaction between learner cognition, learning behavior, and learning environment is influenced by the teacher training environment, which is crucial to enhance the learning engagement of teachers’ online training. The educational meta universe is an online digital space parallel to the real world. In the immersive scenario built by the meta universe, the traditional online and offline complementary relationship of online teacher training is transformed into an integrated relationship of virtual and real integration, which empowers the construction of real situations for teacher training and human-computer collaborative teaching and research with intelligent technology support. For example, teachers can simulate teaching events in real classrooms in the educational meta universe, and test the teaching effectiveness through virtual students’ feedback on teaching activities and teaching methods. For example, teachers can simulate teaching events in the real classroom in the educational meta universe, test the teaching effect through virtual students’ feedback on teaching activities and teaching methods, and help teachers reflect on teaching., The educational meta universe helps teachers to conduct new teaching and research with human-computer collaboration: traditional teaching and research is mostly carried out in the form of workshops and teaching and research groups, which is detached from practice and difficult to attract participating teachers to participate in it. The virtual teachers can be homogeneous or heterogeneous teachers, or they can be set as “expert roles” with guiding role, and they can collaborate with teachers in teaching and research, which changes the new model of teaching and research for the participating teachers. In addition, big data, virtual reality, wearable devices and other intelligent technologies in the educational meta universe support the collection of multimodal data in the teacher training process, and help teacher educators make decisions by measuring key elements of teachers’ cognitive, emotional, behavioral and social interaction learning inputs in the training process through data analysis technology.

### 6.2. Personalized and precise training based on big data, adaptive and other technologies

The inappropriateness of training content and the disconnection from teaching practice are important reasons for the low motivation of teachers’ training. In May 2021, the Ministry of Education and the Ministry of Finance jointly issued the document “Notice on the Implementation of the National Training Program for Primary and Secondary School Kindergarten Teachers (2021–2025),” which emphasizes “To implement a tiered classification of precision training”(The Chinese Ministry of Education, 2021). This indicates that the design of teacher training programs should focus on both the accuracy of the training content and the classification of teachers to improve the relevance and effectiveness of teacher training. Before training, it is necessary to focus on the core needs of teachers’ professional development to make an overall design of training content, rely on big data technology to accurately evaluate teachers’ knowledge and ability foundation, learning preferences, cognitive style and other individual characteristics, and use clustering, text analysis and other technologies to extract and integrate teachers’ professional development needs according to the data indicators (such as browsing time, clicks, downloads, etc.) of teachers in previous online learning platforms, and analyze the personalized needs of participating teachers. In the training process, we can use big data technology to record teachers’ learning behavior data in the training process, such as research results and click data streams and so on. And the training resources are dynamically restructured through adaptive technology, and different types of training resources are organically reorganized to push more targeted learning resources and match the optimal learning path for participating teachers, so as to shift the previous experience-driven training content recommendation to data-driven training resource pushing and improve teachers’ learning efficiency and training quality. After the training, it is necessary to pay attention to both results-oriented and process-oriented evaluation of teachers, follow the data-oriented principle of multi-dimensional evaluation of teachers, and improve the self-efficacy of teacher training.

### 6.3. Establishing a multi-dimensional and three-dimensional online learning support service system

The online learning environment, because of its spatial and temporal separation, is prone to induce a sense of loneliness and alienation among learners, causing them to awaken negative academic emotions such as anxiety and boredom, and hindering their commitment to online learning. Online training tends to focus on intellectual and instrumental support for teachers, but lacks interactive and emotional support for teacher training, and it is necessary to strengthen the construction of the support service system for teacher training in the future to bridge the lack of interactive support and emotional support. On the one hand, the essential feature of interactive support is interaction, and it is necessary to enrich the interaction between teachers and teachers, teachers and the environment, and teachers and teacher educators in the training process and strengthen experiential learning activities. In addition, in the online training, interactive activities such as random selection, linked hands, and online peer evaluation can also strengthen the effective interaction of learners with peers and teachers, enhance the sense of social presence, and help teachers reduce negative academic emotions such as anxiety and fear generated by the sense of isolation. On the other hand, the construction of online learning support service system should pay more attention to emotional support for teachers. Firstly, teacher educators should create a positive and harmonious learning atmosphere for online training, express learning expectations to teachers appropriately, give them effective and immediate feedback, and encourage them to actively participate in interactions to alleviate their anxiety in the training process; Secondly, to enhance the sensitivity of teacher educators, to perceive in a timely manner the intellectual, technical and emotional needs of teachers when participating in training and to provide guidance and assistance, and to reduce teachers’ academic emotions such as anxiety, boredom and boredom due to difficult problems not being solved; Finally, teachers are encouraged to express their own views and give positive responses in the learning exchange to help them enhance their sense of self-achievement, and enhance their pleasant emotional experience in the interaction and emotional exchange with teacher educators and other teachers to meet their psychological needs.

## 7. Implications

This study studied the mechanism of influence of teachers’ academic emotions and motivational beliefs on online learning engagement in online training. Compared with previous studies, first of all, this study enriched the empirical research on teachers’ academic emotions and learning engagement in online training context, and provided empirical data reference for teacher educators to improve the effectiveness of online teacher training. Secondly, previous studies only classified academic emotion from a single dimension of pleasure, while this study classifies academic emotion from the two-dimensional classification standard of pleasure and arousal, enriching the connotation of academic emotion. Finally, this study verifies the effect of academic emotion on its proximal factors from the reverse path, verifies the path hypothesis of the control value theory, and expands the theoretical research of the control value theory.

Although there are some new findings in this study, the following aspects still need further improvement in future studies: first, this study adopted a self-reported approach to measure teachers’ online learning engagement, which may be subjective, and future studies can use multimodal whole process learning data to accurately measure teachers’ online learning engagement; second, the mechanism of negative-high arousal academic emotion on learning engagement is complex, and researchers have also found that it motivates learners to pay more to avoid failure (Zaccoletti et al., 2020), therefore, exactly how negative-high arousal academic emotion affects online learning engagement is to be studied in depth in the future.

## Data availability statement

The raw data supporting the conclusions of this article will be made available by the authors, without undue reservation.

## Author contributions

DZ: Conceptualization, Formal analysis, Methodology, Writing – original draft. SG: Data curation, Formal analysis, Methodology, Writing – original draft. LR: Writing – original draft, Writing – review & editing.

## Funding

This research was supported by funding from the following projects: 2021 Henan Province Higher Education Teaching Reform Research and Practice Project (Degree and Postgraduate Education) (Project No. 2021SJGLX206Y); 2023 Key Projects for Curriculum Reform in Teacher Education in Henan Province (Project No. A010); 2020 National Natural Science Youth Fund Program (Project No. 72004055).

## Conflict of interest

The authors declare that the research was conducted in the absence of any commercial or financial relationships that could be construed as a potential conflict of interest.

## Publisher’s note

All claims expressed in this article are solely those of the authors and do not necessarily represent those of their affiliated organizations, or those of the publisher, the editors and the reviewers. Any product that may be evaluated in this article, or claim that may be made by its manufacturer, is not guaranteed or endorsed by the publisher.
